# Using Mobile Phones to Improve Vaccination Uptake in 21 Low- and Middle-Income Countries: Systematic Review

**DOI:** 10.2196/mhealth.7792

**Published:** 2017-10-04

**Authors:** Clare Oliver-Williams, Elizabeth Brown, Sara Devereux, Cassandra Fairhead, Isaac Holeman

**Affiliations:** ^1^ Homerton College University of Cambridge Cambridge United Kingdom; ^2^ Cardiovascular Epidemiology Unit Department of Public Health & Primary Care University of Cambridge Cambridge United Kingdom; ^3^ Gonville and Caius College University of Cambridge Cambridge United Kingdom; ^4^ University College London London United Kingdom; ^5^ Trinity College University of Cambridge Cambridge United Kingdom; ^6^ Queens’ College University of Cambridge Cambridge United Kingdom; ^7^ King’s College University of Cambridge Cambridge United Kingdom; ^8^ Judge Business School University of Cambridge Cambridge United Kingdom; ^9^ Global Health Academy University of Edinburgh Edinburgh United Kingdom; ^10^ Medic Mobile San Francisco, CA United States

**Keywords:** cell phones, vaccination, communication, telemedicine, mHealth, global health

## Abstract

**Background:**

The benefits of vaccination have been comprehensively proven; however, disparities in coverage persist because of poor health system management, limited resources, and parental knowledge and attitudes. Evidence suggests that health interventions that engage local parties in communication strategies improve vaccination uptake. As mobile technology is widely used to improve health communication, mobile health (mHealth) interventions might be used to increase coverage.

**Objective:**

The aim of this study was to conduct a systematic review of the available literature on the use of mHealth to improve vaccination in low- and middle-income countries with large numbers of unvaccinated children.

**Methods:**

In February 2017, MEDLINE (Medical Literature Analysis and Retrieval System Online), Scopus, and Web of Science, as well as three health organization websites—Communication Initiative Network, TechNet-21, and PATH—were searched to identify mHealth intervention studies on vaccination uptake in 21 countries.

**Results:**

Ten peer-reviewed studies and 11 studies from white or gray literature were included. Nine took place in India, three in Pakistan, two each in Malawi and Nigeria, and one each in Bangladesh, Zambia, Zimbabwe, and Kenya. Ten peer-reviewed studies and 7 white or gray studies demonstrated improved vaccination uptake after interventions, including appointment reminders, mobile phone apps, and prerecorded messages.

**Conclusions:**

Although the potential for mHealth interventions to improve vaccination coverage seems clear, the evidence for such interventions is not. The dearth of studies in countries facing the greatest barriers to immunization impedes the prospects for evidence-based policy and practice in these settings.

## Introduction

In 2005, the World Health Organization encouraged member states to take action to incorporate eHealth in health systems and services. The term electronic health (eHealth) refers to the practice of supporting health care through information and communication technologies; eHealth initiatives have been recognized for their potential to strengthen health systems and to improve access to care [1].

The subset of eHealth initiatives that make use of mobile phones or any portable electronic devices with software applications are often discussed using the term mobile health (mHealth). Mobile technologies have been applied to a diverse range of initiatives outlined in recent reviews of mHealth interventions globally [2,3] and in low- and middle-income countries (LMIC) [4]. Given that nearly 100% of the world’s population lives within reach of a mobile phone signal, many regard mHealth initiatives as particularly promising in LMIC, where other forms of communication infrastructure are underdeveloped [4]. In areas where phone ownership among the general population remains relatively low, community health workers can be key players in mHealth. Equipped with mobile phones, they can efficiently and effectively disseminate information, such as clinical updates, learning resources, and reminders, both to other health workers and to patients [5,6].

Various mHealth interventions in LMIC have aimed to improve vaccination uptake by increasing awareness of vaccine availability and providing timely reminders of when they are due. Vaccination averts approximately 2 to 3 million deaths annually and can be highly cost-effective [7]. Disparities in vaccine coverage persist because of limited resources, vaccines stock outs, geographic inaccessibility and long wait times, and poor health system management in general [8,9]. Additional demand-side barriers relate to parental knowledge and attitudes, fear of side effects, and conflicting priorities [9]. An estimated 18.7 million infants worldwide did not receive routine vaccinations such as the DPT3 (diphtheria) vaccine in 2014, and over 60% of these children live in just 10 LMIC. Evidence suggests that top-down communication strategies are detrimental to some vaccination drives in LMIC, whereas interpersonal communication incorporating local leaders and networks and utilizing a wide range of communication channels are more successful [10]. As mobile technology is widely used to improve health communication in general, mHealth interventions might be used to improve vaccine coverage.

Although the potential for mHealth interventions to improve vaccination coverage seems clear, the evidence for such interventions is not. The global population of unvaccinated children is highly concentrated in a small number of countries; as a result, literature reviews of mobile technology for immunization globally, or even of LMIC in general, may be of limited relevance. To the best of our knowledge, there has been no systematic overview of mHealth for immunization programs in countries with the greatest need to improve vaccination coverage. For this reason, the objective of this systematic review was to summarize the outcomes and implementation challenges of mHealth for vaccination interventions, focusing on 21 countries with high proportions of unvaccinated children [11].

## Methods

### Data Sources and Search Strategy

This systematic review was conducted using a predefined protocol and in accordance with the preferred reporting items for systematic reviews and meta-analyses (PRISMA) and meta-analysis of observational studies in epidemiology (MOOSE) checklists (Multimedia Appendices 1 and ). A literature review was conducted on February 23, 2017 (date last searched) using MEDLINE (Medical Literature Analysis and Retrieval System Online), Scopus, and Web of Science databases. Gray and white literature was also identified on the Communication Initiative Network, TechNet-21, and PATH websites. Search terms were grouped into three categories: those relating to vaccination, such as inoculation and immunization; mHealth, for example, mobile phone or telemedicine; and geographical location. No restrictions were placed on language. Details of the search terms are located in Multimedia Appendix 3. Titles and then abstracts were searched, potentially relevant papers were read, and those that did not meet the predefined inclusion criteria were removed. The inclusion criteria specified the country (Angola, Cambodia, Democratic Republic of the Congo, Ethiopia, India, Indonesia, Iraq, Kenya, Mali, Malawi, Nepal, Niger, Nigeria, Pakistan, the Philippines, Senegal, South Africa, Tanzania, Uganda, Zambia, and Zimbabwe); any form of mhealth (including mobile phone calls, phone apps, text messages, Internet, and email); and an outcome pertaining to vaccination (including uptake of vaccinations, attendance at vaccination appointments, and completeness of vaccination protocol for individuals or for regions). Reference lists of the selected studies and relevant reviews were also searched for additional publications.

### Study Selection and Eligibility

Prospective interventional and observation studies that evaluated mHealth interventions on any part of a vaccination program were of interest if they were based in the relevant countries listed previously. These countries were chosen, as they include the 10 countries where more than 60% of children were unvaccinated for the final dose of Diphtheria-tetanus-pertussis vaccination as of 2014 [11], in addition to 11 countries that also have low routine vaccination uptake where the authors identified ongoing large-scale mHealth initiatives.

### Data Extraction

Data were extracted by 4 authors and a predesigned data abstraction form was used. Any conflicts over inclusion were resolved by discussion. Relevant information included location, age of participants, study design, numbers included in the study, type of mobile phone intervention and frequency, duration of the study, outcome measures, and results. Where multiple publications from the same study were found, only the most up-to-date or comprehensive information was extracted.

### Risk of Bias

The quality of peer-reviewed studies was rated for the risk of bias. Randomized control trials (RCTs) were assessed using the Cochrane Collaboration tool [12]. This tool considers seven different scales: random sequence generation, allocation concealment, blinding of participants and personnel, blinding of outcome assessment, incomplete outcome data, selective reporting, and other bias. Observational studies were evaluated using the Newcastle-Ottawa Scale [13], which uses a star system to assess three aspects: participant selection, comparability of study groups, and ascertainment of outcomes. Studies that received a score of nine stars were judged to be at low risk of bias, studies that scored seven or eight stars were medium risk, and those that scored six or less were at high risk.

### Analysis

Descriptive summary tables were constructed to display the results. Due to the small number and heterogeneity of studies identified, it was not possible to either conduct a statistical analysis of the results or assess publication bias through funnel plots.

## Results

### Studies Identified

**Figure 1:**
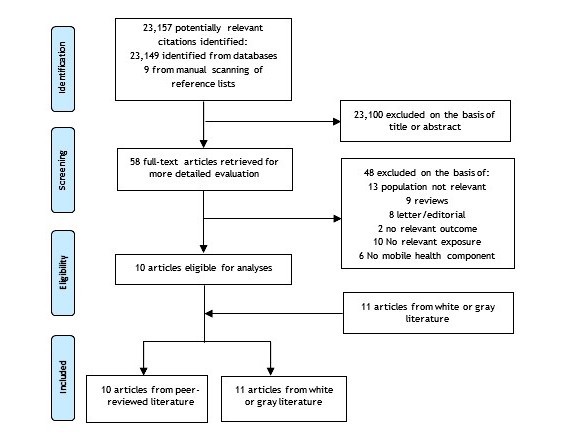
Flowchart for literature search.

The literature search identified 23,157 potentially relevant citations. After screening titles and abstracts, 58 peer-reviewed papers remained for further evaluation, and following detailed assessment, a further 48 were excluded (Figure 1). The remaining 10 unique papers, plus 11 studies from the gray and white literature, were included within this review.

### Characteristics of Included Studies

Of the 21 studies fulfilling the inclusion or exclusion criteria, 10 were peer-reviewed, of which 3 were RCTs. Tables 1 and 2 outline the key characteristics of studies included in this review, and Table 3 summarizes geographical locations. The interventions evaluated in these papers ranged from SMS messages sent to families to remind and encourage them to take their children to the health center for vaccinations, to using mobile phones to record which settlements have been covered by vaccination campaigns, to mobile phone apps helping health workers to update and access relevant data to facilitate vaccination campaigns.

**Table 1:** Summary of relevant papers from peer-reviewed literature.

Lead author, date	Location	Year of study	Age range or mean age	Intervention	Outcome evaluated	Number of participants	Results
Bangure et al, 2015 [14]	Kadoma City, Zimbabwe	2013	Median age of mothers: 26 (intervention group) and 27 (controls)	SMS reminders to attend vaccination appointments at 6, 10, and 14 weeks. SMS sent 7, 3, and 1 day before appointment. Control group received routine health education only.	Percentage of children fully vaccinated with 3 doses of polio, pentavelent, and pneumococcal vaccines at 6, 10, and 14 weeks. Percentage delayed in receiving the 3 vaccines.	304 (152 intervention and 152 controls)	Vaccination coverage was greater in the intervention group (*P*<.001 for all): 6 weeks: 96.7% (147/152) versus 82.2% (125/152); 10 weeks: 96.1% (146/152) versus 80.3% (122/152); 14 weeks: 94.7% (144/152) versus 75.0% (114/152). Controls had a greater delay in vaccination (% delayed, median and interquartile range (IQR) delay): 6 weeks: intervention: 7.2% (11/152), 0 days (0-0), control: 76.3% (116/152), 2 days (0-6) 10 weeks: intervention: 13.2% (20/152), 0 days (0-0), control: 82.9% (126/152), 5 days (2-9) 14 weeks: intervention: 17.8% (27/152), 0 days (0-0), control: 92.1% (140/152), 10 days (6-17).
Brown et al, 2016 [15]	Ibadan, Nigeria	2012-2013	Children aged 0-12 months	Parents were randomly allocated to receive phone calls about vaccination 2 days and 1 day before the appointment, or usual care (no reminder).	Routine vaccination completion at 12 months (1 Bacillus Calmette-Guérin [BCG] dose, 4+ oral polio vaccine doses, 3 diphtheria doses, 3 hepatitis B doses, 1 measles, and yellow fever dose).	605 eligible children	The intervention group was 72% (relative risk 1.72, CI 1.50-1.98) (146 of 148 children in the intervention group vs 86 of 150 children in the control group) more likely to complete vaccination than controls who did not receive calls.
Uddin et al, 2016 [16]	Dhaka (urban) and Sunamgonj (rural), Bangladesh	2013-2014	Pregnant women, mothers with children, 0-11 months	SMS reminders sent to mothers about upcoming vaccination sessions 1 day before, at opening time, and 2 hours before closing time on the day of the vaccination.	Full vaccination rates: 1 dose of BCG; 3 doses of pentavalent (Penta) vaccine at 6, 10, and 14 weeks; and 1 dose of Measles, Mumps, and Rubella (MMR) vaccine at 9 months.	2078 children	Odds ratio (OR) for being fully vaccinated in rural areas: OR 3.6 (95% CI 1.5-8.9). OR for being fully vaccinated in urban areas: OR 2.3 (95% CI 1.1-5.5).
Garcia-Dia et al, 2016 [17]	Bago City area, Philippines	2013-2014	Parents of children aged 12-14 months	Participants were sent either a plain text message (SMS) or a text message with pictures once 7-10 days before the scheduled appointment date. Controls were given a verbal reminder.	MMR vaccination coverage rate Timely vaccination (difference between scheduled date of appointment visit and actual date of visit that the child was brought in for vaccination)	75 parents	Vaccination rates did not differ between the groups. Compared with verbal reminders, text reminders were associated with well-timed vaccination (difference between scheduled date of appointment and actual date of vaccination), average delay: 0.96 days for plain text reminders, 2.72 days for picture text reminders, and 20.64 days for verbal reminders (*P*=.07)
Crawford et al, 2014 [18]	Balaka District, Malawi	2011-2013	Mean age of child: 4 months	Health messages (including vaccination reminders) delivered through pushed SMS and voice messages sent to personal phones and voice messages retrieved from a community phone.	Delivery success rates for the three delivery methods User experience assessed by phone survey: Acceptability, comprehension, and self-reported behavior change.	2611 caregivers of children. A total of 1137 caregivers responded to the phone survey.	Choice of delivery system: retrieved voice messaging 63.35% (1654/2611); Pushed SMS 28.07% (733/2611); Pushed voice message 8.58% (224/2611) Delivery success: Pushed SMS 64.10% (13,053/20,363); Pushed voice 53.81% (1515/2815); Retrieved voice 27.36% (14,455/52,829). Phone survey results: 22.6% (51/226) reported not receiving any messages, most were pushed voice enrollees. 98.9% (263/266) trusted messages they received; 75.2% (200/266) recalled last message. Pushed SMS enrollees were more likely to report intended or actual change in behavior (91%, 87/96) than pushed (56%, 17/30,) or retrieved (65.7%, 92/140,) voice enrollees; *P*=.01.
Kazi et al, 2014 [19]	Karachi, Pakistan	2012-2013	Not given (NG)	SMS messages sent to caregivers to monitor coverage of polio supplementary immunization activities (SIAs): 1. Did the vaccinator visit your home? 2. Did [child] receive polio vaccine? US $0.20 of phone credit was given for replying. Nonresponders were contacted via direct phone calls.	Proportion of caregivers who replied to the SMS or follow-up phone calls. Estimates of vaccine coverage achieved during polio SIAs obtained through automated SMS and currently used methods for estimating vaccine coverage, as utilized by the World Health Organization.	Across 7 districts, 5880 randomly sampled caregivers of a child <5 years	Response rate: first SMS 22.99% (1352/5880); second SMS 14.00% (823/5880). 74.90% (4404/5880) of participants did not respond to SMS messages, of whom, 56.00% (2466/4404) responded to an investigator’s phone calls. Those who responded to calls had similar levels of vaccine coverage to those who responded to SMSs. Reasons given for not responding to SMS (of caregivers who were contactable by direct phone call): “Too busy” 36.01% (888/2466); “Not interested” 32.00% (789/2466); “Unable to read the message” 20.00% (493/2466).
Wakadha et al, 2013 [20]	30 villages within 5 km of Ting’Wan’I hospital in Western Kenya.	2011	Mothers of children up to 4 weeks of age at baseline	Reminder SMS sent (3 days prior and on day of vaccination) for 2 doses of pentavalent vaccination. If the child was vaccinated on time, the mother was given approximately US $2. If the child was not vaccinated, another reminder was sent.	(1) Percentage of children vaccinated at hospital or other health facilities. (2) Percentage who did not receive SMSs (3) Percentage with mobile phone access 4) Follow-up at 14 weeks: influence of financial reward on vaccination	72 mothers (first dose: 69 sent SMS reminders, 3 not sent, second dose: 44 sent SMS)	(1) First dose: 70% (48/69) vaccinated at Ting’wang’I, Hospital, 10% (7/69) at other hospitals. Second dose: 91% (40/44) vaccinated at TWI hospital, 5% (2/44) at other hospitals. (2) Of the 38% (27/72) not sent SMS, 26% (7/27) vaccinated at TWI, 19% (5/27) at other hospitals, 30% (8/27) not vaccinated, and 26% (7/27) unknown. (3) 26% (19/72) had their own phone, and 74% (53/72) had access to another person’s phone (4) Forty-nine mothers reported reminders influenced their decision to vaccinate.
Touray et al, 2016 [21]	10 states in northern Nigeria	2012-2015	NG	Global positioning system–enabled Android phones were given to vaccination teams and were used to record team tracks.	Settlements covered by vaccination teams during polio campaigns	NG	There was a reduction in chronically missed settlements (those missed in the last 3 campaigns): 2014—5833 settlements, 2015—1257 settlements. There was an increase in the number of missed settlements: 2014—4142, 2015—7008.
Balakrishnan et al, 2016 [22]	Bihar, India	2012-2014	NG	Mobile-based tool for health workers that registers when vaccinations are due and administered, creating electronic records.	Received 1+ tetanus vaccine	512 frontline workers, 19,888 children registered	Coverage in implementation area (95% CI): 79.38% (58.90-80.26) (15,771 children vaccinated of 19,888 registered) Coverage in implementation area in the previous year (%): 74.12 Coverage in rest of Bihar (%): 80
Mbabazi et al, 2015 [23]	Kenya: 8 districts of Nairobi and 3 from Nyanza or western provinces	2012	Children aged 9-59 months	A Web-enabled mobile phone app recording house visits (3 days prior and 4 days after vaccination campaigns), vaccinations, and relaying information to campaign organizers.	Percentage of households aware of the campaign before start; Percentage planning to vaccinate their children Post campaign: Percentage of households with children vaccinated against measles; Percentage with a confirmed vaccination.	164,643 houses (161,695 children) pre campaign; 175,617 houses (180,493 children) post campaign	56.00% (92,200/164,643) of households had heard about the campaign. 75.00% (123,482/164,643) of households planned to bring their children for vaccination. 96.00% (168, 592/175,617) of households reported children having had a measles vaccination post campaign, and 92.00% (161,568/175,617) of households had children with a confirmed vaccination.

Eleven initiatives were identified from gray and white literature; eight took place in India, two in Pakistan, and one in Zambia. Several programs involved more than one intervention, including messages sent to parents to encourage their children to get vaccinated, information about vaccination made freely accessible via mobile phone, tools to identify unvaccinated children with the health authority using SMS, data management tools for health workers (such as electronic vaccination records and a mobile phone app to track where vaccinations have been administered and control supplies), and tools to help health workers persuade hesitant families.

### SMS Reminders for Vaccinations

Eight peer-reviewed studies reported the use of phone calls or SMS reminders for vaccinations, two of which additionally offered cash incentives. The three studies that did not offer cash incentives included an RCT by Bangure et al [14] conducted in Zimbabwe. SMS reminders were sent to parents (n=152) when their baby was 6, 10, and 14 weeks old, in addition to routine health education. The control group received health education alone (n=152). At all three time points, the percentage of children fully vaccinated with the relevant dose of polio, pentavalent, and pneumococcal vaccines was significantly higher in the intervention than the control group (<.001), and the delay in receiving the vaccinations was significantly less in the intervention than the control group (<.001). Another RCT by Brown et al [15] conducted in Nigeria identified increased coverage rates relative to the usual care when receiving phone call reminders 2 days and 1 day before a vaccination appointment (Relative risk 1.72, 95% CI 1.50-1.98). Uddin et al [16] similarly found that SMS reminders increased the odds of vaccination uptake in both urban and rural areas; odds ratio (OR) 2.3 (95% CI 1.1-5.5) and OR 3.6 (95% CI 1.5-8.9), respectively. Garcia-Dia et al [17] assessed coverage rates in an RCT in the Philippines after 75 parents were sent either a plain text message (short service message, SMS), a text message with pictures, or a verbal reminder. Although vaccination rates did not differ by reminder, text reminders with and without a picture were associated with a shorter delay in receiving the vaccination than verbal reminders. Crawford et al [18] sent SMS or voice messages to either the personal or community phone of 2611 caregivers of children under the age of 1 year. Pushed SMS messaging (where a message is sent to a phone’s notification center or status bar) was the most successful mode of delivery (64.10%, 13,053/20,363, of sent messages were received). However, most women did not own a mobile phone, so similar numbers of messages were delivered by retrieved voicemail to community phones and pushed SMS to personal phones. No control group was included, but the majority of individuals who received messages trusted (98.8%; 263/266) and could recall (72.2%; 200/277) those messages.

**Table 2:** Summary of studies from white and gray literature.

Name of study or source of participants	Location	Year of study	Age range or mean age	Intervention form	Intervention period and regularity of intervention	Outcome evaluated	Total number of participants	Results
MIRA channel [24]	Haryana, India	2012-ongoing	Children	Integrated mobile phone channel with health information to women and connecting them with health services.	Continuous	Vaccination rates	Not given (NG)	Increase in vaccination rates by 41% (from 51% to 92%, overall rates in Haryana: 78%).
Mobile Kunji [25]	Bihar, India	2011-2015	NG	When a health worker dials the number, they can play a health message—voiced by a character called Dr Anita, an engaging but authoritative female doctor—to the family via their mobile phone.	NG	Percentage of children unvaccinated, Percentage of children (6-11 months) receiving DPT2 (diphtheria) vaccine, Percentage of children (<11 months) with a vaccination card	NG	Mobile Kunji was not found to significantly alter vaccination uptake [26].
UNICEF, India’s National Immunization Day [27]	India	1999-2000	Children	India’s national telecom authority agreed to replace the ringtone with a recorded message reminding the public about the date of the National Immunization Day.	Annually	Number of children vaccinated for polio, Percentage of coverage (2+ doses), Percentage of zero doses, number of polio cases	NG	151 million children vaccinated, 98.6% coverage (at least 2 doses), 0.7% of children with zero doses, 265 cases of polio in 2000.
Mobilink [28,29]	Pakistan	2009-2012	Children	Subscribers to the Mobilink mobile operator will be able to report areas and children where the polio vaccination teams have not reached. The respective health authority will then be in contact to vaccinate the missed children. Mobilink also sends an SMS to create awareness about polio.	Period: 1-3 days, with >3 rounds for reporting unvaccinated children.	NG	NG	15,000 SMS messages about unvaccinated children were received during February 15-17, 2010.
Aarogyam [30]	Uttar Pradesh, India	2008 onwards	Children under 5 years	Health alerts are sent to parents about vaccination through an SMS and phone calls.	NG	Vaccination coverage	NG	Vaccination coverage has shown a significant positive trend over time. Polio, Bacillus Calmette-Guérin (BCG), measles, and tetanus coverage has gone up from approximately 60% in 2008 to 91% in 2010.
Khushi Baby [31]	Northern India	2015	Infants	Electronic copy of the vaccination record stored on a necklace. Health workers scan the necklace using an app on their mobile phone to transfer vaccination data to the necklace. Data are also automatically uploaded to “the cloud.” Parents get vaccination reminder voice calls.	Continuous	NG	NG	Pilot study is ongoing.
mSakhi [32]	Uttar Pradesh, India	2011	NG	A mobile-based interactive multimedia learning app for health workers.	4 months	Increase in knowledge in maternal-newborn care (including vaccination)	25 health visitors	Qualitative data indicated improved counseling during home visits and increased credibility of health workers in the community.
HealthPhone [33]	India	2009-ongoing	Children	Video reference library that covers vaccination and SMS messages for those who cannot access video.	No time limit, continuous	Multiple health outcomes, including uptake of vaccines	NG	“After we put HealthPhone into the hands of village women...their health and the health of their children dramatically improved.”
Freedom Polio [34]	India	2012	Children under 5 years	An app that allows health works to track where polio vaccinations have been administered.	No time limit, continuous	NG	21 million children	NG
UNICEF, Zambian Health Ministry, two mobile phone companies, Zain and Mobile Telephone Networks [35]	28 districts in Zambia	2009	Children under 5 years	SMS: “Your child can be healthier! Take your children under age five to the nearest health centre for free vaccinations from 20-25 July.”	NG	NG	NG	NG
Interactive Research and Development’s (IRD) Interactive alerts [36,37]	Karachi, Pakistan	2012 onwards	NG	Mobile phone–based vaccine registry system that uses SMS reminders to caregivers and conditional cash transfers to caregivers and health workers.	NG	Vaccination coverage and timeliness	14,000 infants	Interim data analysis suggests improved immunization coverage and timeliness; an impact evaluation study is underway to assess this more thoroughly.

**Table 3:** Geographical locations of the included studies.

Location	Number of studies included
Bangladesh	1
India	9
Kenya	2
Malawi	1
Nigeria	2
Pakistan	3
The Philippines	1
Zambia	1
Zimbabwe	1

### Cash Incentives to Increase Vaccination Uptake

Two studies used cash incentives to increase vaccination uptake while sending SMS reminders. Kazi et al [19] used SMS messages sent to 5880 caregivers in Pakistan, along with a conditional cash transfer in the form of approximately US $0.20 of phone credit, to monitor polio vaccination coverage. Response rates to the SMS messages were low (74.90%, 4404/5880, of participants did not respond). The initial nonresponders who were followed up by phone call had similar rates of vaccination uptake to those who responded to the SMS messages. Wakadha et al [20] conducted a pilot study exploring the feasibility of setting up an integrated mobile phone–based system to remind and incentivize mothers (n=72) to vaccinate their children in rural Kenya. Mothers received SMS reminders of vaccination dates and conditional cash transfers of either mPESA (a mobile-based money transfer service) credit or phone credit, if the child was vaccinated within 4 weeks of the scheduled date. The small sample size and lack of a comparison group meant that it was not possible to draw conclusions about the program’s effectiveness, but enrolled mothers reported mostly positive experiences at the end of the study, and most mothers did have access to a phone. Importantly, this study was limited by its focus on a single facility. Caregivers who took children to nearby facilities for vaccinations were recorded as unvaccinated by the first facility and thereafter, were not sent additional SMS.

### Mobile-Based Interactive Apps for Health Workers

Three studies used mobile-based interactive learning apps to aid or track the progress of health workers in vaccination. Touray et al [21] utilized the global positioning system of Android phones to track where vaccination teams had been, which helped reduce the number of settlements in northern Nigeria that were not covered in the last three campaigns from 5833 in 2014 to 1257 in 2015. Balakrishnan et al [22] found no improvement in coverage for tetanus when health workers used a mobile phone tool that created electronic vaccination records and registered when vaccination was due and administered. Mbabazi et al [23] evaluated a mobile phone app used by health workers that was designed to assess the awareness and intention to take part in a measles vaccination campaign before the campaign’s onset at house visits, as well as to evaluate the uptake of the vaccinations after the campaign. Of the more than 150,000 households included in the survey, approximately half were aware of the vaccination campaign, and once informed, 74.99% (123,482/164, 643) of households planned to bring their children in for vaccination. After the campaign, 95.99% (168,592/175,617) of households reported their child had received a measles vaccination, and 92.00% (161, 568 of 175,617) had this independently confirmed. This intervention was found to reduce misconceptions about vaccination, and the use of the mobile phone app to assess uptake of vaccination helped inform service delivery plans.

### Risk of Bias

Of the 10 peer-reviewed studies, three studies did not evaluate controls or individuals unexposed to the intervention, so it was not possible to evaluate their risk of bias using the Newcastle-Ottawa Scale or the Cochrane Collaboration tool. In the eight studies that could be evaluated, the risk of selection bias affecting the results was judged to be low for one RCT by Bangure et al but with a higher risk of bias in the other two RCTs (Table 4). The risk of bias in the observational studies was also deemed to be high, with most of the concern regarding the possibility of outcome bias and bias arising from a lack of comparability (Table 5).

**Table 4:** Assessment of bias in randomized controlled trials.

Paper	Risk of bias						
	Random sequence generation	Allocation concealment	Blinding of participants and personnel	Blinding of outcome assessment	Incomplete outcome data	Selective reporting	Other bias
Bangure et al [14]	Low	Low	Unclear	Unclear	Low	Low	Low
Brown et al [15]	Low	Low	High	Medium	Low	Low	Low
Garcia-Dia et al [17]	Low	Medium	High	Unclear	Low	Low	Low

**Table 5:** Assessment of bias in observational cohort studies.

Paper	Selection^a^	Comparability^b^	Outcome or exposure^c^
Uddin et al [16]	2	2	2
Wakadha et al [20]	4	0	1
Touray et al [21]	3	0	3
Balakrishnan et al [22]	2	0	1

^a^Maximum score is 4.

^b^Maximum score is 2.

^c^Maximum score is 3.

### Initiatives Identified From White and Gray Literature

Of the eleven initiatives identified, six showed some evidence of impact on vaccination rates. Implementation of the MIRA channel [24] (an integrated mobile phone channel providing health information to women and connecting them with health services) corresponded with a 41% increase in vaccination rates. A program using the Mobile Kunji program [25,26] (in which the health worker can play a health message to the family via their mobile phone) recorded a 5% decrease in the percentage of children (6-11 months) unvaccinated with the first diphtheria vaccine and a 6% increase in children receiving the second diphtheria vaccine. Another successful strategy included the involvement of India’s national telecom authority who replaced the dial tone on mobile phones with a recorded message that reminded the public of National Immunization Day [27], whereas the Mobilink mobile operator in Pakistan recorded 13,000 SMS messages about unvaccinated children during the annual polio vaccination campaign in 2010 and circulated seven million SMS reminders in 2009 [28,29]. Aarogyam [30] reported improved vaccination uptake through the use of automatic voice calls and SMS reminders sent to parents about vaccination appointments, among other postnatal care.

Unfortunately, other initiatives did not provide quantitative results. Some pilot studies are ongoing (Khushi Baby [31]), and one provided qualitative evidence of improved knowledge [32]. No outcomes were found for three studies, one study used educational videos accessible on mobile phones [33], another looked at an app to track vaccinations [34], and two studies used SMS vaccination reminders [35-37].

## Discussion

Overall, mHealth technology can and has been used to increase vaccination uptake in LMIC, but the quality of the evidence is limited, and further research is needed to better quantify its potential impact and to determine the most effective strategies.

### Evidence That mHealth Interventions Can be Effective in Increasing Vaccination Uptake

The literature reviewed indicates that mobile technology can be used in a variety of ways to improve vaccination uptake. Although most studies lacked comparison groups, the results broadly suggest an improved uptake of vaccinations with mobile phone–based interventions.

SMS reminders for vaccination appointments were found to increase uptake and reduce delays in receiving vaccinations in Zimbabwe [14]. In Kenya, mothers who received SMS reminders about vaccination appointments reported mostly positive experiences [23]. A decrease in the percentage of unvaccinated children and an increase in the number of children with a vaccination card were found when health care workers used their mobile phones to play a prerecorded message to families. Furthermore, a 41% increase in vaccination rates was observed in rural India after the introduction of an integrated mobile channel providing health information and connecting mothers with health services [24].

However, some studies reported no improvement upon intervention. A study from Pakistan found low response rates to SMS messages about vaccinations, even when a financial reward was attached [19].

### Challenges in the Use of mHealth Interventions to Increase Vaccination Uptake

The studies we reviewed, as well as the related research that these studies cited to explain the design of their interventions, raise a number of challenges that can impede the integration of mobile phones into vaccination programs. Several of these studies discuss rates of phone ownership in their particular areas of intervention, including how these differ among men and women (Crawford et al, Uddin et al, and Kazi et al). In LMIC, generally, women are 21% less likely to own a mobile phone than men (increasing to 37% in Asia) [38]. As women are the primary caregivers to children, this may impact mHealth vaccination interventions. Furthermore, two-thirds of illiterate adults are women [39], which can further reduce the effectiveness of SMS messages. In households where the father owns the mobile phone, it is imperative that the father is engaged in the project, as exemplified in the study by Wakadha et al [20], where in a few cases husbands did not approve of the study.

Frequent exposure to SMS messages can result in the effectiveness of the message being weakened; in a different setting, Strandbygaard et al found that participants stopped reading reminder messages after a few weeks [40]. Therefore, the effectiveness of messages of different length and over time needs to be assessed when sending SMSs.

Developing the appropriate infrastructure [9] and ensuring adequate resources are available is important. Weaknesses in other areas of the health system may render mHealth interventions aiming to increase demand for services meaningless: mHealth can improve access to vaccines only as long as they remain consistently available from health centers [9]. Additionally, increasing demand for vaccination can have unintended consequences. One study reported that extensive and comprehensive communication campaigns for 15 new vaccines led to greater demand for vaccination in a number of LMIC. However, high demand resulted in vaccine shortages, which later thwarted the increased demand [41]. For this reason, policy makers and implementers of mHealth interventions to improve vaccination programs should be aware that eHealth interventions in general [42], and mHealth interventions in particular [6,43] are deeply complex and context-dependent.

### Limitations, Opportunities, and Need for Further Research

Of the literature reviewed, the included studies were predominantly observational studies that appraised process and usage output. These had various methodological limitations such as (1) sample selection based on convenience, without randomization; (2) small sample sizes; (3) lack of information on process validation, including recruitment type, response rate, and retention rate; and (4) no control groups. These limitations make the conclusions of the observational studies less secure. Given the potential of mHealth, RCTs in LMIC to determine the efficacy of using mHealth for vaccinations are needed.

It is clear that there are a number of gaps in the literature concerning this topic in the countries of interest. Relatively more compelling evidence exists for mHealth interventions addressing demand-side barriers to service uptake, whereas fewer evaluated interventions aim to boost immunization by strengthening health systems through data management, decision support, or provider training and education. This is a notable gap because reviews of why children go unvaccinated document not only highlight gaps in household knowledge and attitudes but also issues related to poor service quality and accessibility [8,9]. Moreover, there is reason to believe that this gap can be addressed because outside immunization programs, mHealth interventions have been widely used as strengthening tools for health systems [4]. For example, given that a study on stock tracking of malaria medications in Tanzania showed a 52% reduction in medication stock-outs within 21 weeks of the induction of weekly SMS requesting stock counts [44], there is a precedent for the integration of mHealth into vaccination stock control.

### Conclusions

There is reason to be optimistic regarding the potential for mobile phones to increase vaccination coverage in LMIC. Mobile technologies are flexible and widely available tools that can be utilized in myriad ways. This review provides evidence of potential effectiveness for SMS reminders to families regarding vaccination, as well as for educational tools for health workers.

However, the research is preliminary and limited. Further research is needed to determine the most effective mHealth interventions and to refine their use, for example, clarifying the optimal schedule of reminders for programs using SMS reminders of vaccination appointments. It will also be necessary to evaluate different mHealth interventions against each other, and against other potential programs, to examine their comparative cost-effectiveness at increasing vaccination coverage. mHealth interventions addressing vaccination stock-outs, cold storage, or other health systems strengthening challenges merit further study.

Overall, there is preliminary evidence to support the use of mHealth technology to increase vaccination coverage in LMIC. However, further research is needed to guide and improve the use of these technologies in the future and to strengthen the case for their cost-effectiveness.

## Appendix

Multimedia Appendix 1 PRISMA 2009 checklist.

Multimedia Appendix 2 MOOSE checklist.

Multimedia Appendix 3 Search terms used to identify relevant published literature.

## Abbreviations

eHealth: electronic health
LMIC: low- and middle-income countries
mHealth: mobile health
OR: odds ratio
RCT: randomized controlled trial
