# Supplementary material for: Using Mobile Phones to Improve Vaccination Uptake in 21 Low- and Middle-Income Countries: Systematic Review
Source: JMIR Mhealth Uhealth. 2017 Oct 4;5(10):e148. doi: 10.2196/mhealth.7792 (PMC5647459; doi:10.2196/mhealth.7792)
Supplement: Multimedia Appendix 1 [file mhealth_v5i10e148_app1.pdf]

Appendix 1. PRISMA 2009 check-list

| Section/topic | # | Checklist item | Reported on page # |
|---|---|---|---|
| **TITLE** | | | |
| Title | 1 | Identify the report as a systematic review, meta-analysis, or both. | 1 |
| **ABSTRACT** | | | |
| Structured summary | 2 | Provide a structured summary including, as applicable: background; objectives; data sources; study eligibility criteria, participants, and interventions; study appraisal and synthesis methods; results; limitations; conclusions and implications of key findings; systematic review registration number. | 2 |
| **INTRODUCTION** | | | |
| Rationale | 3 | Describe the rationale for the review in the context of what is already known. | 3 |
| Objectives | 4 | Provide an explicit statement of questions being addressed with reference to participants, interventions, comparisons, outcomes, and study design (PICOS). | 3 |
| **METHODS** | | | |
| Protocol and registration | 5 | Indicate if a review protocol exists, if and where it can be accessed (e.g., Web address), and, if available, provide registration information including registration number. | Appendix |
| Eligibility criteria | 6 | Specify study characteristics (e.g., PICOS, length of follow-up) and report characteristics (e.g., years considered, language, publication status) used as criteria for eligibility, giving rationale. | 4 |
| Information sources | 7 | Describe all information sources (e.g., databases with dates of coverage, contact with study authors to identify additional studies) in the search and date last searched. | 4 |
| Search | 8 | Present full electronic search strategy for at least one database, including any limits used, such that it could be repeated. | Appendix |
| Study selection | 9 | State the process for selecting studies (i.e., screening, eligibility, included in systematic review, and, if applicable, included in the meta-analysis). | 4 |
| Data collection process | 10 | Describe method of data extraction from reports (e.g., piloted forms, independently, in duplicate) and any processes for obtaining and confirming data from investigators. | 4 |
| Data items | 11 | List and define all variables for which data were sought (e.g., PICOS, funding sources) and any assumptions and simplifications made. | 4 |
| Risk of bias in individual studies | 12 | Describe methods used for assessing risk of bias of individual studies (including specification of whether this was done at the study or outcome level), and how this information is to be used in any data synthesis. | 4 |
| Summary measures | 13 | State the principal summary measures (e.g., risk ratio, difference in means). | n/a |
| Synthesis of results | 14 | Describe the methods of handling data and combining results of studies, if done, including measures of consistency (e.g., $I^2$) for each meta-analysis. | n/a |


| Section/topic | # | Checklist item | Reported on page # |
|---|---|---|---|

| | | | |
|---|---|---|---|
| Risk of bias across studies | 15 | Specify any assessment of risk of bias that may affect the cumulative evidence (e.g., publication bias, selective reporting within studies). | 4 |
| Additional analyses | 16 | Describe methods of additional analyses (e.g., sensitivity or subgroup analyses, meta-regression), if done, indicating those which were pre-specified. | n/a |
| **RESULTS** | | | |
| Study selection | 17 | Give numbers of studies screened, assessed for eligibility, and included in the review, with reasons for exclusions at each stage, ideally with a flow diagram. | 6 and Figure 1 |
| Study characteristics | 18 | For each study, present characteristics for which data were extracted (e.g., study size, PICOS, follow-up period) and provide the citations. | 6, Tables 1-2 |
| Risk of bias within studies | 19 | Present data on risk of bias of each study and, if available, any outcome level assessment (see item 12). | 8, Tables 4-5 |
| Results of individual studies | 20 | For all outcomes considered (benefits or harms), present, for each study: (a) simple summary data for each intervention group (b) effect estimates and confidence intervals, ideally with a forest plot. | n/a |
| Synthesis of results | 21 | Present results of each meta-analysis done, including confidence intervals and measures of consistency. | n/a |
| Risk of bias across studies | 22 | Present results of any assessment of risk of bias across studies (see Item 15). | n/a |
| Additional analysis | 23 | Give results of additional analyses, if done (e.g., sensitivity or subgroup analyses, meta-regression [see Item 16]). | n/a |
| **DISCUSSION** | | | |
| Summary of evidence | 24 | Summarize the main findings including the strength of evidence for each main outcome; consider their relevance to key groups (e.g., healthcare providers, users, and policy makers). | 9-10 |
| Limitations | 25 | Discuss limitations at study and outcome level (e.g., risk of bias), and at review-level (e.g., incomplete retrieval of identified research, reporting bias). | 9-10 |
| Conclusions | 26 | Provide a general interpretation of the results in the context of other evidence, and implications for future research. | 9-10 |
| **FUNDING** | | | |
| Funding | 27 | Describe sources of funding for the systematic review and other support (e.g., supply of data); role of funders for the systematic review. | 11 |

Appendix 2. MOOSE Checklist

The use of mobile phones to improve vaccination in low and middle income countries: a systematic review

| Criteria | | Brief description of how the criteria were handled in the meta-analysis |
|---|---|---|
| **Reporting of background should include** | | |
| √ | Problem definition | Mobile technology (mHealth) may be used to engage local parties in communication strategies to improve vaccination uptake in low and middle income countries. However, the extent of evidence for this unclear. Thus, this systematic review aims to draw together the evidence for and against mHealth interventions in vaccine coverage employed in low and middle income countries. |
| √ | Hypothesis statement | mHealth interventions can improve vaccination uptake in low and middle income countries |
| √ | Description of study outcomes | Vaccination uptake, coverage and knowledge about vaccination |
| √ | Type of exposure or intervention used | Any mHealth intervention; one that uses mobile phone technology |
| √ | Type of study designs used | Observation studies and randomized controlled trials |
| √ | Study population | Low and middle income countries with low levels of vaccinated children |
| **Reporting of search strategy should include** | | |
| √ | Qualifications of searchers | The credentials of the investigators are indicated in the authors list. |
| √ | Search strategy, including time period included in the synthesis and keywords | Search strategy and time periods are detailed in page 4 of the manuscript and in Appendix 3. |
| √ | Databases and registries searched | MEDLINE, Scopus and Web of Science, and three health organization websites; the Communication Initiative Network, TechNet-21, and PATH |
| √ | Search software used, name and version, including special features | We did not employ a search software. Mendeley was used to merge retrieved citations and eliminate duplications. |
| √ | Use of hand searching | We hand-searched bibliographies of retrieved papers and relevant reviews for additional references. |
| √ | List of citations located and those excluded, including justifications | Details of the literature search process are outlined in the flow chart. Citations for the included studies are within the reference list. The citation list for excluded studies is available upon request. |
| √ | Method of addressing articles published in languages other than English | We placed no restrictions on language. No articles in languages other than English were identified. |
| √ | Method of handling abstracts and unpublished studies | No authors were contacted. |
| √ | Description of any contact with authors | n/a |
| **Reporting of methods should include** | | |
| √ | Description of relevance or appropriateness of studies assembled for assessing the hypothesis to be tested | Detailed inclusion and exclusion criteria are described in the Methods section. |
| √ | Rationale for the selection and coding of data | Data extracted from each of the studies were relevant to the population characteristics, study design, exposure, and outcome. |
| √ | Assessment of confounding | Confounding was assessed when evaluating within study bias |
| √ | Assessment of study quality, including blinding of quality assessors; stratification or regression on possible predictors of study results | No sensitivity analyses were conducted due to the small number and heterogeneous nature of the studies identified |
| √ | Assessment of heterogeneity | N/A |
| √ | Description of statistical methods in sufficient detail to be replicated | N/A |
| √ | Provision of appropriate tables and graphics | We included 5 main tables |
| **Reporting of results should include** | | |
| √ | Graph summarizing individual study estimates and overall estimate | N/A |
| √ | Table giving descriptive information | Tables 1-2 |

| Criteria | | Brief description of how the criteria were handled in the meta-analysis |
|---|---|---|
| | for each study included | |
| √ | Results of sensitivity testing | N/A |
| √ | Indication of statistical uncertainty of findings | N/A |
| **Reporting of discussion should include** | | |
| √ | Quantitative assessment of bias | No quantitative assessment of bias was possible, but a qualitative discussion is included. |
| √ | Justification for exclusion | We excluded studies that did not use an mHealth intervention or did not assess any aspect of vaccine coverage, or studies that were conducted in countries other than those of interest |
| √ | Assessment of quality of included studies | We discussed the between-study differences and risk of bias in the discussion. |
| **Reporting of conclusions should include** | | |
| √ | Consideration of alternative explanations for observed results | We discussed the limitations of the observational studies and how they may call into question how tenable the findings are. |
| √ | Generalization of the conclusions | The generalisability is limited by the small number of identified studies in countries of interest, which is mentioned in the discussion. |
| √ | Guidelines for future research | We discuss the challenges of integrating mobile technology into vaccination programs, and avenues for future research in the discussion. |
| √ | Disclosure of funding source | This is located in the abstract |

Appendix 3: Search terms used to identify relevant peer-reviewed literature

| Category | Search Terns |
|---|---|
| Immunisation | “Disease prevention” OR “Disease protection” OR “Disease control” OR “Inject*” OR “Immunity” OR “GVAP” OR “global vaccine action plan” OR “Immuniz*” OR “Immunis*” OR “Vaccin*” OR “Inoculat*” OR “Jab*” OR “Shot*” OR "Vaccines" OR "Immunization" OR "Vaccination" OR "Immunity" OR "Injections" OR "Communicable Disease Control") |
| mHealth | “Virtual health” OR “Mobile *phone*” OR “Cell *phone*” OR "Cell Phones" OR “Mobile technolog*” OR “Mobile Application*” OR "Mobile Applications" OR “Mobile device*” OR “App” OR “Apps” OR “Module*” OR “SMS messag*” OR “Smartphone*” OR “M-health” OR “mHealth” OR “m Heath” OR “mobileHealth” OR “mobile-health” OR “mobile health” OR “e-Health” OR “eHealth” OR “e health” OR “electronichealth” OR “electronic-health” OR “electronic health” OR “telehealth” OR “tele-health” OR “telemedicine” OR “tele-medicine” OR "Telemedicine" OR “telepharmacy” OR “telecommunication*” OR "Telecommunications |
| Location | “Nepal” OR “India” OR “Indonesia” OR “Cambodia” OR “Kenya” OR “Ethiopia” OR “Tanzania” OR “Democratic Republic of Congo” OR “DRC” OR “Mali” OR “Malawi” OR “Uganda” OR “Senegal” OR “Zambia” OR “Angola” OR “Niger” OR “Zimbabwe” OR “Iraq” OR “Nigeria” OR “Pakistan” OR “Philippines” OR “South Africa” |
