# Supplementary material for: Using Mobile Phones to Improve Vaccination Uptake in 21 Low- and Middle-Income Countries: Systematic Review
Source: JMIR Mhealth Uhealth. 2017 Oct 4;5(10):e148. doi: 10.2196/mhealth.7792 (PMC5647459; doi:10.2196/mhealth.7792)
Supplement: Multimedia Appendix 2 [file mhealth_v5i10e148_app2.pdf]

Appendix 2. MOOSE Checklist

The use of mobile phones to improve vaccination in low and middle income countries: a systematic review

Criteria		Brief description of how the criteria were handled in the meta-analysis
**Reporting of background should include**		
√	Problem definition	Mobile technology (mHealth) may be used to engage local parties in communication strategies to improve vaccination uptake in low and middle income countries. However, the extent of evidence for this unclear. Thus, this systematic review aims to draw together the evidence for and against mHealth interventions in vaccine coverage employed in low and middle income countries.
√	Hypothesis statement	mHealth interventions can improve vaccination uptake in low and middle income countries
√	Description of study outcomes	Vaccination uptake, coverage and knowledge about vaccination
√	Type of exposure or intervention used	Any mHealth intervention; one that uses mobile phone technology
√	Type of study designs used	Observation studies and randomized controlled trials
√	Study population	Low and middle income countries with low levels of vaccinated children
**Reporting of search strategy should include**		
√	Qualifications of searchers	The credentials of the investigators are indicated in the authors list.
√	Search strategy, including time period included in the synthesis and keywords	Search strategy and time periods are detailed in page 4 of the manuscript and in Appendix 3.
√	Databases and registries searched	MEDLINE, Scopus and Web of Science, and three health organization websites; the Communication Initiative Network, TechNet-21, and PATH
√	Search software used, name and version, including special features	We did not employ a search software. Mendeley was used to merge retrieved citations and eliminate duplications.
√	Use of hand searching	We hand-searched bibliographies of retrieved papers and relevant reviews for additional references.
√	List of citations located and those excluded, including justifications	Details of the literature search process are outlined in the flow chart. Citations for the included studies are within the reference list. The citation list for excluded studies is available upon request.
√	Method of addressing articles published in languages other than English	We placed no restrictions on language. No articles in languages other than English were identified.
√	Method of handling abstracts and unpublished studies	No authors were contacted.
√	Description of any contact with authors	n/a
**Reporting of methods should include**		
√	Description of relevance or appropriateness of studies assembled for assessing the hypothesis to be tested	Detailed inclusion and exclusion criteria are described in the Methods section.
√	Rationale for the selection and coding of data	Data extracted from each of the studies were relevant to the population characteristics, study design, exposure, and outcome.
√	Assessment of confounding	Confounding was assessed when evaluating within study bias
√	Assessment of study quality, including blinding of quality assessors; stratification or regression on possible predictors of study results	No sensitivity analyses were conducted due to the small number and heterogeneous nature of the studies identified
√	Assessment of heterogeneity	N/A
√	Description of statistical methods in sufficient detail to be replicated	N/A
√	Provision of appropriate tables and graphics	We included 5 main tables
**Reporting of results should include**		
√	Graph summarizing individual study estimates and overall estimate	N/A
√	Table giving descriptive information	Tables 1-2

Criteria		Brief description of how the criteria were handled in the meta-analysis
	for each study included	
√	Results of sensitivity testing	N/A
√	Indication of statistical uncertainty of findings	N/A
**Reporting of discussion should include**		
√	Quantitative assessment of bias	No quantitative assessment of bias was possible, but a qualitative discussion is included.
√	Justification for exclusion	We excluded studies that did not use an mHealth intervention or did not assess any aspect of vaccine coverage, or studies that were conducted in countries other than those of interest
√	Assessment of quality of included studies	We discussed the between-study differences and risk of bias in the discussion.
**Reporting of conclusions should include**		
√	Consideration of alternative explanations for observed results	We discussed the limitations of the observational studies and how they may call into question how tenable the findings are.
√	Generalization of the conclusions	The generalisability is limited by the small number of identified studies in countries of interest, which is mentioned in the discussion.
√	Guidelines for future research	We discuss the challenges of integrating mobile technology into vaccination programs, and avenues for future research in the discussion.
√	Disclosure of funding source	This is located in the abstract
