# Supplementary material for: Using Mobile Phones to Improve Vaccination Uptake in 21 Low- and Middle-Income Countries: Systematic Review
Source: JMIR Mhealth Uhealth. 2017 Oct 4;5(10):e148. doi: 10.2196/mhealth.7792 (PMC5647459; doi:10.2196/mhealth.7792)
Supplement: Multimedia Appendix 3 [file mhealth_v5i10e148_app3.pdf]

Appendix 3: Search terms used to identify relevant peer-reviewed literature

Category	Search Terns
Immunisation	“Disease prevention” OR “Disease protection” OR “Disease control” OR “Inject*” OR “Immunity” OR “GVAP” OR “global vaccine action plan” OR “Immuniz*” OR “Immunis*” OR “Vaccin*” OR “Inoculat*” OR “Jab*” OR “Shot*” OR "Vaccines" OR "Immunization" OR "Vaccination" OR "Immunity" OR "Injections" OR "Communicable Disease Control")
mHealth	“Virtual health” OR “Mobile *phone*” OR “Cell *phone*” OR "Cell Phones" OR “Mobile technolog*” OR “Mobile Application*” OR "Mobile Applications" OR “Mobile device*” OR “App” OR “Apps” OR “Module*” OR “SMS messag*” OR “Smartphone*” OR “M-health” OR “mHealth” OR “m Heath” OR “mobileHealth” OR “mobile-health” OR “mobile health” OR “e-Health” OR “eHealth” OR “e health” OR “electronichealth” OR “electronic-health” OR “electronic health” OR “telehealth” OR “tele-health” OR “telemedicine” OR “tele-medicine” OR "Telemedicine" OR “telepharmacy” OR “telecommunication*” OR "Telecommunications
Location	“Nepal” OR “India” OR “Indonesia” OR “Cambodia” OR “Kenya” OR “Ethiopia” OR “Tanzania” OR “Democratic Republic of Congo” OR “DRC” OR “Mali” OR “Malawi” OR “Uganda” OR “Senegal” OR “Zambia” OR “Angola” OR “Niger” OR “Zimbabwe” OR “Iraq” OR “Nigeria” OR “Pakistan” OR “Philippines” OR “South Africa”
